# The Envelope Gene of Hepatitis B Virus Is Implicated in Both Differential Virion Secretion and Genome Replication Capacities between Genotype B and Genotype C Isolates

**DOI:** 10.3390/v9040062

**Published:** 2017-03-28

**Authors:** Haodi Jia, Yanli Qin, Chaoyang Chen, Fei Zhang, Cheng Li, Li Zong, Yongxiang Wang, Jiming Zhang, Jisu Li, Yumei Wen, Shuping Tong

**Affiliations:** 1 Key Lab of Medical Molecular Virology, School of Basic Medical Sciences, Fudan University, Shanghai 200032, China; hdjia14@fudan.edu.cn (H.J.); 13111010065@fudan.edu.cn (C.C.); 13817459252@126.com (F.Z.); 15211010047@fudan.edu.cn (C.L.); zongli1226@163.com (L.Z.); boldawing@aliyun.com (Y.W.); ymwen@shmu.edu.cn (Y.W.); 2 Department of Infectious Diseases, Huashan Hospital, Fudan University, Shanghai 200032, China; qyl2122@hotmail.com (Y.Q.); jmzhang@fudan.edu.cn (J.Z.); 3 Liver Research Center, Rhode Island Hospital, Warren Alpert School of Medicine, Brown University, Providence, RI 02903, USA; ji_su_li_md@brown.edu

**Keywords:** *core* gene, *envelope* gene, genome replication, genotype, hepatitis B virus, virion secretion

## Abstract

Chronic infection by hepatitis B virus (HBV) genotype C is associated with a prolonged replicative phase and an increased risk of liver cancer, compared with genotype B infection. We previously found lower replication capacity but more efficient virion secretion by genotype C than genotype B isolates. Virion secretion requires interaction between core particles and ENVELOPE proteins. In the present study, chimeric constructs between genotype B and genotype C clones were generated to identify the structural basis for differential virion secretion. In addition to dimeric constructs, we also employed 1.1mer constructs, where the cytomegalovirus (CMV) promoter drove pregenomic RNA transcription. Through transient transfection experiments in Huh7 cells, we found that exchanging the entire *envelope* gene or just its *S* region could enhance virion secretion by genotype B clones while diminishing virion secretion by genotype C. Site-directed mutagenesis established the contribution of genotype-specific divergence at codons 108 and 115 in the *preS1* region, as well as codon 126 in the *S* region, to differential virion secretion. Surprisingly, exchanging the *envelope* gene or just its *S* region, but not the *core* gene or 3′ *S* region, could markedly increase intracellular replicative DNA for genotype C clones but diminish that for genotype B, although the underlying mechanism remains to be clarified.

## 1. Introduction

The hepatitis B virus (HBV) causes acute and chronic infection of the liver, with the latter being a global public health problem because of its widespread distribution and severe sequelae, including liver cirrhosis and hepatocellular carcinoma (HCC) [1,2]. HBV can be classified into ten genotypes according to the nucleotide sequence divergence of its genomic DNA [3,4,5,6]. Genotypes B and C are responsible for the majority of chronic HBV infections in East Asian countries. Chronic HBV infection is a dynamic process involving the interaction between the virus, hepatocytes, and immune cells. Numerous epidemiological studies suggest that genotype C is more pathogenic than genotype B due to a prolonged hepatitis B e antigen (HBeAg) positive phase characterized by a higher viral load [7,8,9,10,11,12]. Moreover, adulthood infection with genotype C has a greater risk of becoming chronic [13]. On the other hand, genotype B infection is associated with a higher risk of fulminant hepatitis and acute exacerbation of chronic infection [14,15,16]. Comparative studies of the biological properties between these two major HBV genotypes could help gain a better understanding of the molecular basis for their different clinical outcomes. In a previous study, we cloned full-length genotype B and genotype C genomes from Chinese and U.S. patients. Transient transfection of these HBV genomes into Huh7 cells, a human hepatoma cell line, revealed that most genotype C clones (or isolates) replicated less efficiently than genotype B clones (or isolates) but possessed higher virion secretion efficiency [17].

In this regard, HBV is an enveloped virus with a relaxed circular DNA (rcDNA) genome of 3.2 kb. The rcDNA is converted to covalently closed circular (ccc) DNA in the nucleus of infected hepatocytes, which serves as a transcriptional template [18]. Four genes are arranged in a circular and overlapping manner; *precore/core*, *polymerase (P)*, *preS1/preS2/S (envelope)*, and *X*, which generate seven viral proteins through alternative translation initiation from the *precore/core* and *preS1/preS2/S* genes. Thus, HBeAg and CORE protein are products of the *precore/core* gene and *core* gene alone, respectively. Translation initiation from the *preS1*, *preS2*, and *S* region AUG codons in the *envelope* gene generate LARGE (L: *preS1/preS2/S*), MEDIUM (M: *preS2/S*), and SMALL (S: *S* domain alone) ENVELOPE proteins, respectively. Expression of the seven viral proteins is ensured by the transcription of four size forms of co-terminal RNAs of 3.5 kb (HBeAg, CORE, and P), 2.4 kb (L), 2.1 kb (M and S), and 0.7 kb (HBx) [18]. The 3.5-kb RNAs are over genome length and hence terminally redundant. Only the 3.5-kb pregenomic RNA (pgRNA) is required for genome replication. First, it serves as mRNA for both CORE and P proteins. Second, it is packaged together with the P protein inside the core particle assembled from the CORE protein [19,20]. Genome replication as catalyzed by the P protein involves minus strand DNA synthesis using pgRNA as the template, followed by pgRNA degradation and plus strand DNA synthesis using the minus stranded DNA as a template. L and S proteins are essential for virion secretion but play distinct functions [21,22]. The L protein interacts with core particles and retains the S protein towards the formation of the 42-nm virions. Otherwise the default function of the S protein is release as the 22-nm noninfectious subviral particles. Subviral particles constitute the bulk of hepatitis B surface antigen (HBsAg) and can reach 10,000- to 1,000,000- fold higher concentration than virions in the blood of infected individuals [1,21].

In the present study, we attempted to identify the structural basis for differential virion secretion efficiencies between clones of genotype B and genotype C. As virion formation requires the interaction between core particles and ENVELOPE proteins, we focused our attention on the *core* and *envelope* genes. Cloning of the full-length HBV genome to a vector disrupts continuity in the HBV genome and prevents transcription of the terminally redundant pgRNA. In our original study, we used a recircularized HBV genome or a tandem dimer of the HBV genome cloned to a pUC18 vector via the unique *SphI* site (*SphI* dimer) for transfection experiments [17]. Considering that differential replication capacities between clones of the two HBV genotypes complicate data interpretation, in the present study we also cloned 1.1 copies of the HBV genome (the DNA equivalent of pgRNA) to pcDNA3.1zeo (−) vector to overproduce the pgRNA through the exogenous cytomegalovirus (CMV) promoter [23]. We hoped that such a 1.1mer construct would show much higher replication capacity than SphI dimer, thus increasing virion secretion. Moreover, similar levels of the pgRNA would diminish the difference in the replication capacities between the two genotypes, thus simplifying data interpretation.

## 2. Materials and Methods

### 2.1. The 1.1mer over-Length HBV Construct and SphI Dimer for Genome Replication

HBV clones 22.5 (GenBank accession number: KU964112) and 24.6 (KU964143) of genotype B, as well as 17.3 (KU964036) and 27.2 (KU964186) of genotype C have been previously described [17]. Clone 56 (AF100309) of genotype B was a kind gift from Dr. Youhua Xie, Fudan University. A 1.1mer construct for these clones was generated by inserting nucleotide sequence 1818–3215/1–1932 into the *SacI* and *HindIII* sites of the pcDNA3.1zeo (−) vector in two sequential steps. First, HBV DNA fragment 240–1932 was amplified by polymerase chain reaction (PCR) using *SphI* dimer as the template [17], with a *HindIII* site attached to the antisense primer. The PCR product was doubly digested with *XbaI* and *HindIII*, and the resulting HBV DNA fragment was inserted into the *XbaI*-*HindIII* sites of the pcDNA3.1zeo (−) vector. Next, HBV DNA fragment 1818–3215/1–247 was amplified by PCR and cloned to the *SacI*-*XbaI* sites of the above construct in a similar way (with the *SacI* site attached to the sense primer). Fragment exchange and site-directed mutagenesis were performed on such 1.1mer constructs as detailed below. To study the biological properties of HBV under endogenous promoters, 1.1mer constructs with DNA fragment exchange or site-directed mutagenesis were remade into *SphI* dimer using a newly developed method [24].

### 2.2. DNA Fragment Exchange and Site-Directed Mutagenesis

To exchange the *core* gene (positions 1901–2452) between 1.1mer constructs of the two genotypes, a 1.6-kb chimeric DNA fragment covering positions 1818–3215/1–247 was generated by overlap extension PCR, with positions 1901–2452 replaced. The PCR product was doubly digested with *SacI* and *XbaI* for replacement of the cognate fragment in the 1.1mer construct. To exchange the entire *envelope* gene (position 2848–3215/1–835) or just the *S* region (position 155–835) between the 1.1mer construct, a 2.8-kb chimeric fragment covering positions 1818–3215/1–1406 was generated by overlap extension PCR, doubly digested with *SacI* and *BamHI* (position 1406), and used to replace the cognate *SacI*-*BamHI* fragment in the 1.1mer construct. In a similar way, a chimeric fragment covering positions 247–1406 was generated and doubly digested with *XbaI*-*BamHI*, so as to exchange just the 3′ half of the *S* region (position 430–835) between these two genotypes. Overlap extension PCR was also used to introduce the L108I/T115S mutations (at the amino acid level) into the *preS1* region for genotype B clones, as well as the I108L/S115T changes for genotype C clones, followed by replacement of the 1.6-kb *SacI*-*XbaI* restriction fragment. Similar fragment exchange was used to introduce the A152T mutation (at the nucleotide level) to reduce S protein translation [22]. The T126I mutation in the *S* domain for two genotype B clones, as well as the I126T mutation for two genotype C clones, were introduced by exchange of the *XbaI*-*BamHI* restriction fragment with PCR products. The 4B genome with a nonfunctional encapsidation signal (4B ε^−^) served as a negative control in the transfection experiments. It contains the G1879T/T1880A double mutation in the loop of the ε signal, thus abolishing pgRNA packaging and consequently genome replication [25].

### 2.3. Subgenomic Expression Constructs for *ENVELOPE* Proteins

The 0.7mer HBV DNA construct has a 2.3-kb HBV DNA fragment encompassing nt 2721–3215/1–1770 inserted upstream of the SV40 polyadenylation signal and cloned to the pBluescript vector [22]. The 0.7mer construct used in this study is a chimeric construct with the backbone of clone 4B and the *envelope* gene of clone 6.2. It could express all the three ENVELOPE proteins under endogenous promoters and enhancers but not any other HBV proteins. A stop codon in the *preS2* region (C117A) was used to abolish L and M protein expression [22]. Alternatively, the *preS2* and *S* gene ATGs were mutated to ATA and GCG, respectively, to abolish both M and S protein expression.

### 2.4. Transient Transfection and Measurement of Secreted HBsAg

DNA constructs were purified by the high-speed plasmid midi kit (Macherey-Nagel, Duren, Germany), followed by phenol and chloroform extraction. The human hepatoma cell lines Huh7 and HepG2 were cultured in Dulbecco’s Modified Eagle’s Medium (GIBCO, Grand Island, NY, USA), supplemented with 10% fetal bovine serum (Sigma, St. Louis, MO, USA). Transient transfection was performed on cells seeded in 6-well plates using Lipofectamine 3000 reagent (Invitrogen, Carlsbad, CA, USA), with pUC18 DNA, to bring the total amount of DNA to 2 µg/well. HBsAg secreted to culture supernatant was measured by an enzyme-linked immunosorbent assay (ELISA) kit (KHB, Shanghai, China) with proper dilution (1:100–1:500) to prevent signal saturation.

### 2.5. Detection of HBV DNA Replication and Virion Secretion

**Figure 1:**
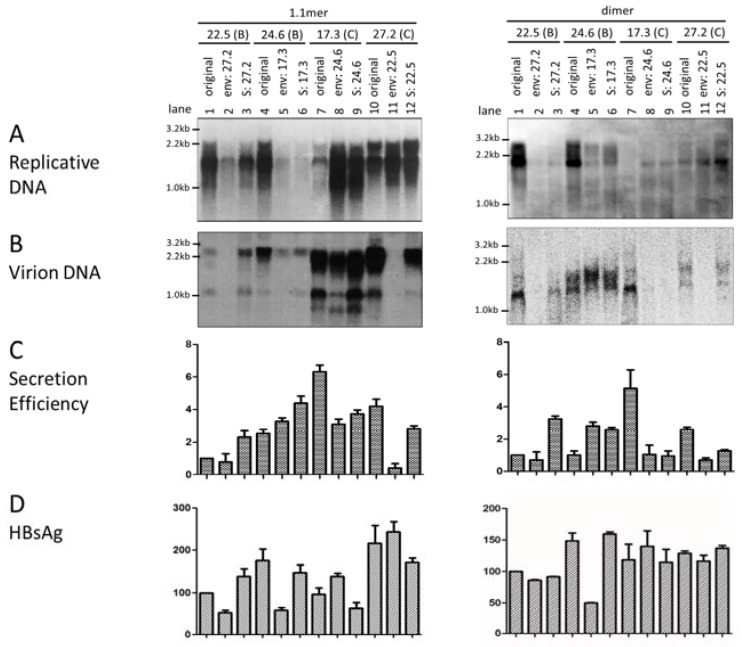
Impact of exchanging the entire *envelope* gene or just the *S* region between clones of genotype B and genotype C on hepatitis B virus (HBV) DNA replication and virion secretion. Both the 1.1mer construct (left panels) and *SphI* dimer (right panels) were used for the exchange of the entire *envelope* (env) gene or just the *S* region. The original and chimeric constructs (2 µg) were transfected to Huh7 cells, which were harvested four days later. (**A**) Southern blot analysis of intracellular replicative DNA (**B**) and extracellular virion-associated DNA from a representative transfection experiment. Mixed probes of genotype B and genotype C were used for hybridization. Positions of the 3.2-kb, 2.2-kb, and 1.0-kb DNA are indicated. (**C**) Ratio of extracellular virion DNA/intracellular replicative DNA. Densitometric values were obtained from Southern blots from three independent transfection experiments, using ImageJ software. The ratio for clone 22.5 was set arbitrarily at 1. (**D**) Secreted hepatitis B surface antigen (HBsAg) averaged from three transfection experiments (1:300 dilution for 1.1mer, 1:500 for dimer), with values for clone 22.5 set arbitrarily at 100.

**Figure 2:**
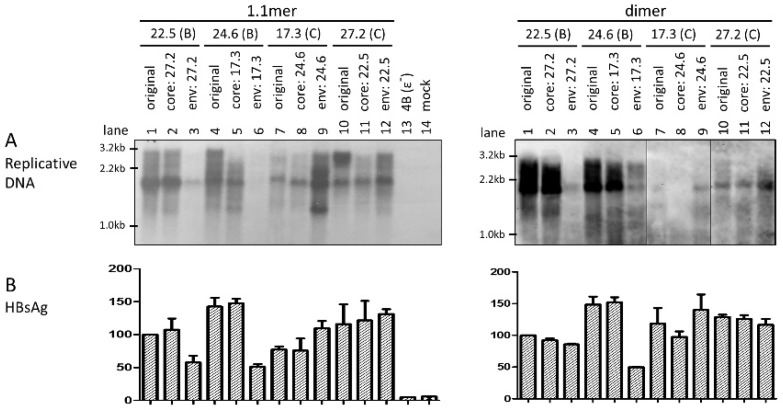
Exchanging the *core* gene between the two HBV genotypes did not alter the intracellular levels of replicative DNA. The *core* gene was exchanged between clones of the two HBV genotypes, as both 1.1mer construct (left panels) and dimer construct (right panels). Chimeras with the *envelope* gene exchanged were analyzed in parallel for comparison. Huh7 cells were transiently transfected with these HBV constructs and harvested four days later. (**A**) Southern blot analysis of replicative DNA from cell lysate using mixed probes of the two genotypes. A genotype A (clone 4B) mutant deficient in pregenomic RNA (pgRNA) encapsidation (ε^−^) served as a negative control. (**B**) Secreted HBsAg averaged from three transfection experiments (1:300 dilution for 1.1mer, 1:500 for dimer), with values for clone 22.5 set arbitrarily at 100.

The details of DNA analysis have been described previously [17,22,26]. Briefly, cells seeded in 6-well plates were harvested at day 4 post-transfection. Core particles were precipitated from half of the cell lysate in lysis buffer containing 0.5% NP40. Virions were immunoprecipitated from 1.4 mL of precleared culture supernatant by a mixture of custom-made polyclonal rabbit anti-preS1 antibody (Genscript, Nanjing, China) (3 µL) and rabbit anti-HBs antibody (Novus, Littleton, CO, USA) (1 µL) precojugated to 10 µL protein G-agarose beads (BioVision, Milpitas, CA, USA). The preS1 antibody targets residues 12–46 (MGTNLSVPNPLGFFPDHQLDPAFGANSNNPDWDFN) and could neutralize HBV infectivity [27,28]. Following nuclease digestion to remove transfected DNA and proteinase K digestion to disrupt core particles or virions, DNA was extracted with phenol and precipitated with ethanol using 10 µg of salmon sperm DNA as carrier. Purified DNA was separated in 1.3% agarose gel with 0.5 µg/mL ethidium bromide added to show the equal loading of carrier DNA for both replicative DNA and virion DNA (data not shown). DNA was transferred to a positively charged nylon membrane (Roche, Indianapolis, IN, USA) for hybridization. The nearly full-length (3.1-kb) HBV DNA for probe making was obtained from a cloned genome of genotype B or C by nested PCR amplification and labeled with [^32^P] dGTP or dCTP by a random primed DNA labeling kit (Roche). Some of the blots for replicative DNA (Figure 1A, right panel; Figure 2A, right panel; Figure 4A; Figure 5A) were hybridized with a digoxigenin-labeled HBV RNA probe, as previously described [28]. Briefly, a 0.7-kb HBV DNA fragment covering positions 1266–1950 was cloned to the *KpnI*-*XhoI* sites of the pcDNA3 vector, and a positive-stranded HBV RNA probe was generated by in vitro transcription of the plasmid linearized at the *XhoI* site using T7 RNA polymerase (DIG-Northern Starter kit from Roche). For quantification, the grey values of signals on the blots were measured by ImageJ software [29].

## 3. Results

### 3.1. Rationale for Using 1.1mer Construct to Compare Virion Secretion between Genotypes B and C

The objective of this study was to identify the structural basis for more efficient virion secretion by genotype C than genotype B. Based on our previous findings [17], we chose two genotype B clones and two genotype C clones for further characterization. Full-length sequencing revealed that both clones 22.5 and 24.6 belong to the B2 subgenotype, while clones 17.3 and 27.2 belong to the C2 and C1 subgenotypes, respectively. Following transient transfection with the *SphI* dimer constructs into Huh7 cells, the level of intracellular replicative DNA was lower for the genotype C clones, especially 17.3 (Figure 1A, right panel). Virion secretion efficiency, as judged by the ratio of extracellular virion DNA/intracellular replicative DNA, was highest for clone 17.3, followed by 27.2 (Figure 1C, right panel). In the present study, we continued to employ the dimeric construct for making some chimeras and site-directed mutants. However, mutagenesis by restriction fragment exchange will revert a dimer into a monomer, necessitating the remaking of a dimer for each new construct. More importantly, the amount of virion DNA produced following transient transfection with *SphI* dimer constructs is rather low, as a consequence of the low replication capacity of genotype C clones. Towards this end, the 1.1mer construct was generated, with sequence 1818–3215/1–1932 inserted to the pcDNA3.1zeo (−) vector. This should lead to robust transcription of pgRNA driven by the CMV promoter [23], thus greatly enhancing HBV DNA replication and, consequently, virion secretion. It was hoped that under the common CMV promoter, a similar level of pgRNA would be transcribed to achieve a comparable level of genome replication and thus simplify the comparison of viron secretion efficiency.

### 3.2. Swapping the Envelope Gene Could Reverse the Replication Phenotype between Clones of the Two Genotypes

As L and S proteins are required for virion formation and release, the entire *envelope* gene or just the *S* region was swapped between clones 22.5 (genotype B) and 27.2 (genotype C) and also between clones 24.6 (genotype B) and 17.3 (genotype C) to evaluate the *envelope* gene as a contributor of differential virion secretion. Surprisingly, the 1.1mer construct of clone 17.3 continued to display a much lower level of replicative DNA than the two genotype B clones. A similar result was obtained when the 1.1mer construct was transiently transfected to HepG2 cells, another human hepatoma cell line supporting HBV genome replication and virion secretion (Supplementary Figure S1). Moreover, replacing its *envelope* gene or just the *S* region with that of clone 24.6 markedly increased replicative DNA (Figure 1A, left panel, compare lanes 7–9). Similar, although less dramatic effects, were observed with *SphI* dimer (Figure 1A, right panel). Conversely, inserting the *envelope* gene or *S* region of clone 17.3 into clone 24.6 drastically reduced DNA replication for both 1.1mer and dimer constructs (Figure 1A, lanes 4–6). Replacing the *envelope* gene of clone 22.5 with that of clone 27.2 also markedly reduced intracellular replicative DNA, whether for the 1.1mer construct or *SphI* dimer (Figure 1A, lanes 1 and 2). Overall, these results revealed the association of the *envelope* gene of genotype C with reduced level of replicative DNA. The impact of the *S* gene on the replication of the 1.1mer construct was further validated in HepG2 cells (Supplementary Figure S2).

### 3.3. Level of Replicative DNA Was Unaffected by Exchanging the Core Gene or 3′ S Region between the Two HBV Genotypes

**Figure 3:**
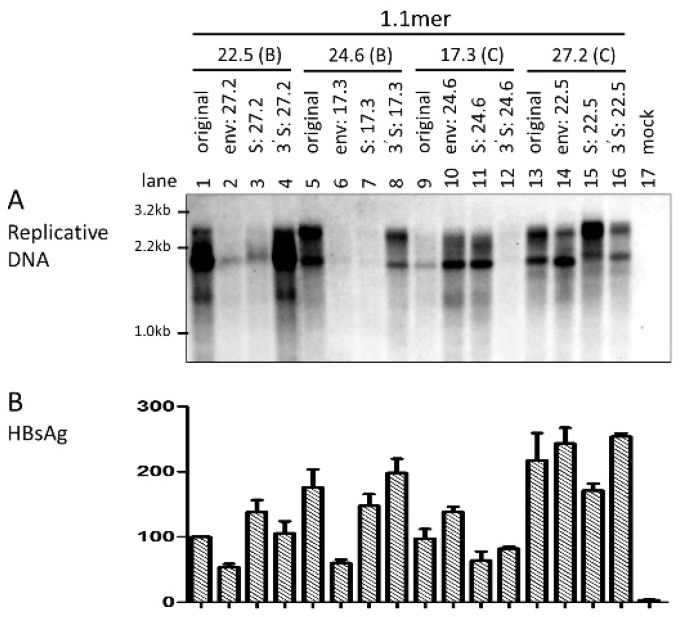
Impact of exchanging the 3′ *S* region on levels of intracellular replicative DNA and extracellular HBsAg. (**A**) The 1.1mer construct was used for exchange of the 3′ *S* region. The original construct and chimeric constructs with the entire *envelope* gene or the entire *S* region replaced were analyzed in parallel. Core particles were extracted from Huh7 cells at day 4 post-transfection for Southern blots of intracellular replicative DNA. Mixed probes of genotype B and genotype C were used for hybridization. (**B**) ELISA for HBsAg measured from culture supernatant from three independent experiments at the dilution of 1:300.

That exchanging the entire *envelope* gene or just the *S* region could markedly alter intracellular level of replicative HBV DNA was quite unexpected. To substantiate this finding, we also exchanged the *core* gene between these two pairs of genotype B—genotype C clones. Remarkably, *core* gene replacement did not reduce replication for the two genotype B clones as either the 1.1mer construct (Figure 2A, left panel) or *SphI* dimer (Figure 2A, right panel). It also failed to increase replication for clone 17.3 of genotype C as a 1.1mer construct. This is in sharp contrast to the constructs with the entire *envelope* gene exchanged, which were transfected in parallel to serve as internal controls (Figure 2A, left panel, lanes 3, 6 and 9; right panel, lanes 3 and 6). In an initial attempt to identify the region responsible for differential replication capacity, we exchanged the 3′ *S* region only. As shown in Figure 3A, such an exchange failed to significantly alter the replication activity for either genotype B or genotype C clones.

### 3.4. Exchanging the Entire Envelope Gene Also Affected HBsAg Level in Culture Supernatant, Especially for 1.1mer Construct

HBsAg was measured from culture supernatant of transfected Huh7 cells by ELISA, and Figure 1D, Figure 2B, and Figure 3B show the relative HBsAg titers averaged from three transfection experiments. Interestingly, replacement of the *envelope*, but not the *core* gene, for the 1.1mer construct resulted in a marked reduction of HBsAg titer for the two genotype B clones but increased the HBsAg titer for clone 17.3 of genotype C (Figure 2B, left panel, lanes 1, 3, 4, 6, 7, and 9). A similar trend was observed for *SphI* dimer, although the effect was striking only for replacement of the *envelope* gene of clone 24.6 by that of clone 17.3 (Figure 2B, right panel, lanes 4 and 6). Exchanging just the *S* region or its 3′ end did not markedly reduce HBsAg titers for the two genotype B clones or increase HBsAg for clone 17.3 of genotype C (Figure 1D and Figure 3B), suggesting that the *preS* region of genotype B confers higher a HBsAg titer.

### 3.5. The Envelope Gene of Genotype C Was Associated with Higher Efficiency of Virion Secretion

Virions were immunoprecipitated from culture supernatant of transfected Huh7 cells by a combination of anti-preS1 and anti-S antibodies, followed by DNA extraction and Southern blot analysis. Due to the drastic difference in intracellular HBV DNA, we quantified the amount of both intracellular replicative DNA and extracellular virion DNA for each construct in the Southern blots, and calculated the ratio of virion DNA/replicative DNA as an indicator of virion secretion efficiency. A typical Southern blot of virion DNA from chimeric constructs of the entire *envelope* gene or just the *S* region is shown in Figure 1B, with the ratios averaged from three separate transfection experiments summarized in Figure 1C. It is evident that clone 17.3 of the C2 subgenotype had the highest rate of virion secretion, followed by clone 27.2 of subgenotype C1. Very similar results were obtained in HepG2 cells (Supplementary Figure S1). Replacing the *envelope* gene or the *S* region of clone 17.3 with that of clone 24.6 markedly reduced virion secretion (Figure 1C, compare lanes 7–9). Conversely, the *envelope* gene or the *S* region from clone 17.3 conferred more efficient virion secretion for clone 24.6 of genotype B (Figure 1C, lanes 4–6). For the pair of 22.5–27.2, the *S* region from clone 27.2 conferred more efficient virion secretion for clone 22.5; conversely the *S* region and especially the entire *envelope* gene from clone 22.5 impaired virion secretion. The impact of the *S* region on virion secretion was also tested and confirmed in HepG2 cells (Supplementary Figure S2C). Replacing the entire *envelope* gene of clone 22.5 with that of clone 27.2 rather reduced the ratio of virion DNA/replicative DNA for both the 1.1mer and dimer constructs (Figure 1C, compare lane 2 with lane 1). In the reverse experiment, replacing the *envelope* gene of clone 27.2 with that of clone 22.5 also impaired virion secretion, even for 1.1mer construct (Figure 1C, lanes 10 and 11).

### 3.6. Two Residues in the preS1 Domain Contributed to Genotypic Difference in Virion Secretion

**Figure 4:**
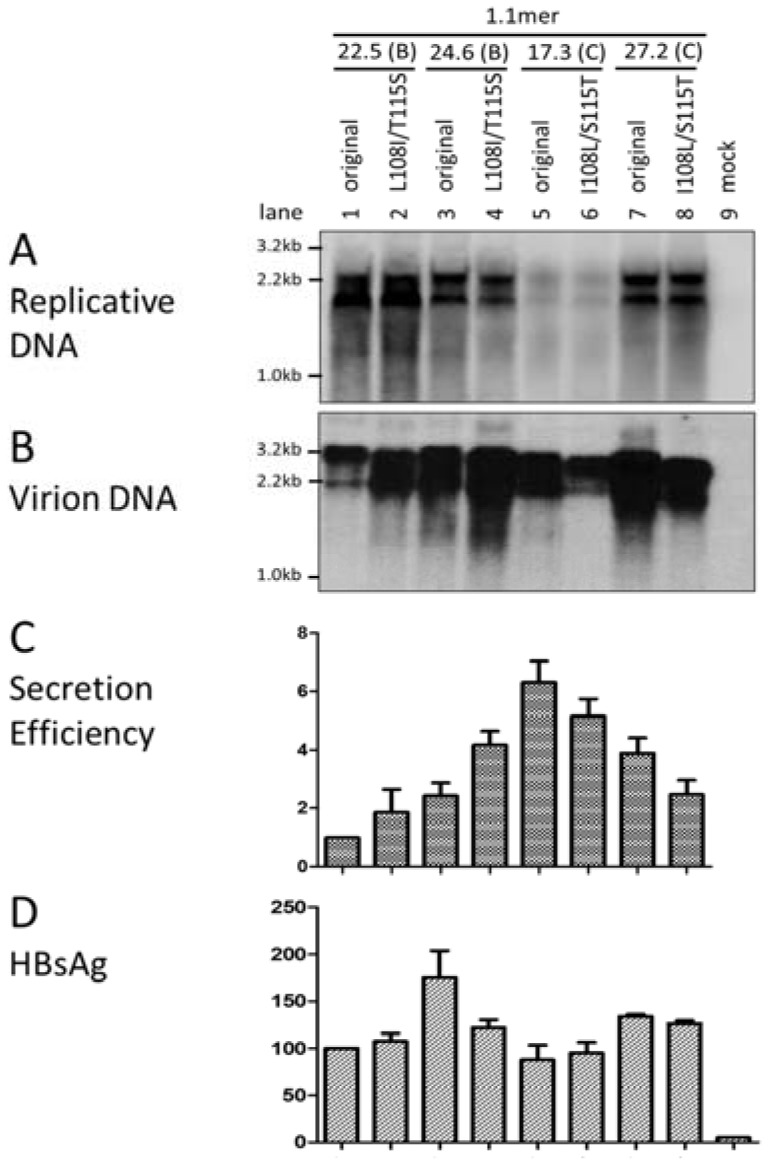
Effect of exchanging two codons in the *preS1* region between the two HBV genotypes on the efficiency of virion secretion. Codons 108 and 115 in the *preS1* region were exchanged between the 1.1mer constructs of genotype B and genotype C clones. Huh7 cells were transiently transfected with the parental constructs and site-directed mutants, and harvested four days later. Southern blot analysis of (**A**) intracellular replicative DNA and (**B**) virion DNA using mixed genotype B/C probes. (**C**) Calculated ratio of extracellular virion DNA/intracellular replicative DNA following densitometric analysis of the Southern blots from three independent transfection experiments. The value for clone 22.5 of genotype B was set arbitrarily at 1. (**D**) Secreted HBsAg values from three independent transfection experiments, with the value for clone 22.5 set arbitrarily at 100. Culture supernatant was diluted 1:300 for ELISA.

The sequence from Arg103 to Ser124 in the *preS* domain of the L protein is highly conserved among different HBV isolates. These 22 amino acid residues are involved in interaction with core particles leading to virion formation [30,31]. Alignment of 2224 sequences of genotype B and 2227 sequences of genotype C available from the HBV database revealed genotype specific variations at two positions within this sequence [32], with most genotype B isolates having L108 (92.6%) and T115 (98.0%), in contrast to I108 (99.7%) and S115 (98.0%) found in genotype C isolates. To establish the impact of these two divergent positions on virion secretion, they were exchanged between clones of the two genotypes in the context of the 1.1mer construct. While the point mutations did not affect genome replication in either Huh7 or HepG2 cells (Figure 4A; Supplementary Figure S2A), the L108I/T115S substitutions increased virion DNA for the two genotype B clones in both cell lines (Figure 4C; Supplementary Figure S2C). Conversely, virion secretion in the two genotype C clones was diminished by the I108L/S115T substitutions. Therefore, the amino acid sequence difference at these two positions in *preS1* domain of L protein at least partly accounts for different virion secretion efficiencies between the two genotypes.

### 3.7. I126 in the S Domain Was Partly Responsible for Efficient Virion Secretion by Genotype C

**Figure 5:**
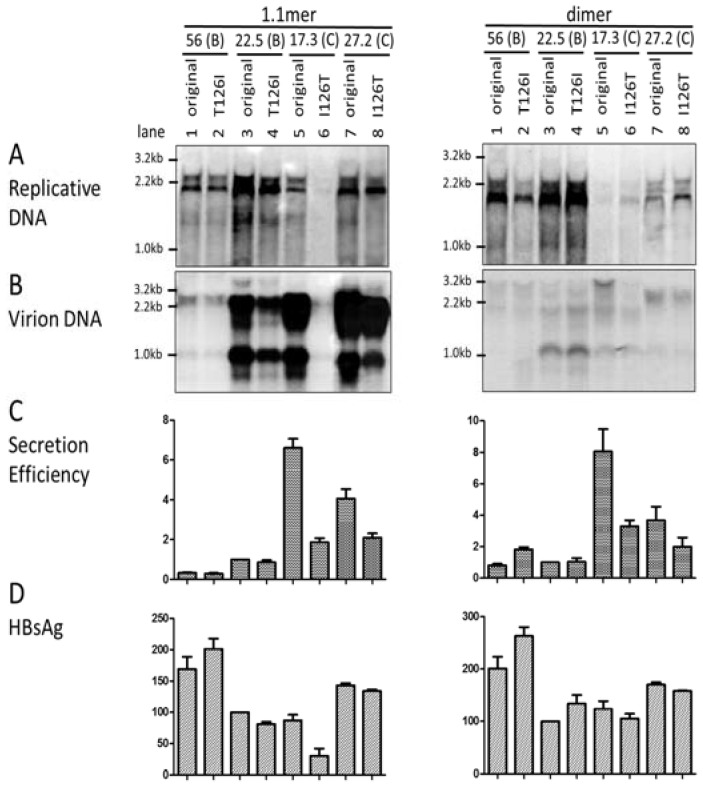
Impact of exchanging codon 126 in the *S* region between genotype B and genotype C clones on HBV virion secretion. The T126I mutation was introduced to two genotype B clones, while the I126T mutation was introduced to two genotype C clones. The parental constructs and the site-directed mutants as 1.1mer construct (left) or *SphI* dimer (right) were transiently transfected to Huh7 cells, which were harvested 4 days later. Shown are Southern blot analysis of (**A**) intracellular replicative DNA and (**B**) virion-associated DNA, (**C**) the ratio of extracellular virion DNA/intracellular replicative DNA as an indicator of secretion efficiency, (**D**) and secreted HBsAg. Culture supernatant was diluted 1:300 (for 1.1mer) or 1:500 (for dimer) for ELISA.

While most HBV genotypes, including genotype B, have T126 in the *S* domain, genotype C is unusual in having I126 as its wild-type sequence [3]. Considering the large difference in chemical properties between threonine and isoleucine, we explored the possible contribution of this genotype-specific position to different virion secretion capacities. The T126I change failed to significantly alter virion secretion for the 1.1mer construct of the two genotype B clones (56 and 22.5) analyzed, although it moderately increased virion secretion for the *SphI* dimer of clone 56 (Figure 5B). Introducing the I126T substitution reduced virion secretion from both the 1.1mer and dimer constructs of the two genotype C clones, especially clone 17.3 (Figure 5B, C). With only one exception (1.1mer construct of clone 22.5), I126 was associated with a higher HBsAg titer than T126 (Figure 5D).

### 3.8. The L/S Protein Ratio Produced by the 1.1mer Construct Appeared Optimal for Virion Secretion

L protein could inhibit HBsAg secretion, and a too high or too low L/S protein ratio diminishes virion secretion [33,34]. Efficient genome replication achieved by the 1.1mer construct makes it the preferred HBV DNA construct for stable transfection into human hepatoma cell lines to generate virus particles for infection experiments [35,36]. Considering transcriptional interference among co-terminal HBV RNAs [28], we wondered whether overproduction of the 3.5-kb pgRNA from the 1.1mer construct suppresses transcription of the 2.4-kb subgenomic RNA to reduce L protein expression, thus diminishing virion secretion. In this regard, we previously found that an A152T mutation affecting the −3 position of the *S* gene translation initiation site could reduce HBsAg secretion from a 1.5mer construct of genotype A by about 70% without impairing virion secretion [22].

**Figure 6:**
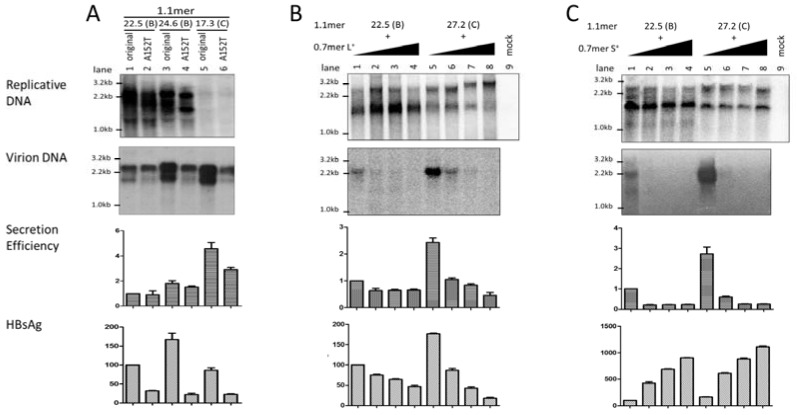
Impact of reducing endogenous S protein expression or providing extra L or S protein to HBV virion secretion from the 1.1mer construct. (**A**) The A152T mutation was introduced to clones of genotype B or genotype C to reduce S protein expression. Both the parental constructs and site-directed mutants were transfected to Huh7 cells. (**B**) Huh7 cells in 6-well plates were transfected with 1 µg of the 1.1mer genotype B or genotype C clone, together with 0, 0.125, 0.25, or 0.5 µg of 0.7mer expression construct for L protein. Variable amounts of pUC18 DNA were added to make the total amount of DNA be 2 µg. (**C**) Huh7 cells in 6-well plates were transfected with 1 µg of the 1.1mer genotype B or genotype C clone, together with 0, 0.25, 0.5, or 0.75 µg of 0.7mer expression construct for S protein, as well as variable amounts of pUC18 DNA for a total of 2 µg. For all three panels, shown from top are intracellular replicative DNA, virion DNA, calculated virion secretion efficiency (from three transfection experiments), and secreted HBsAg (averaged from three transfection experiments; 1:300 dilution for panel A; 1:100 dilution for panel B; 1:500 dilution for panel C).

Introducing the A152T mutation into the 1.1mer constructs did reduce the amount of HBsAg secreted for both genotype B and genotype C clones (Figure 6A, bottom panel). However, virion secretion was diminished as well (Figure 6A, 2nd and 3rd panels). In another approach, Huh7 cells were co-transfected with fixed amount of 1.1mer construct of clone 22.5 or clone 27.2 and an increasing amount of the 0.7mer expression construct for L protein (Figure 6B) or S protein (Figure 6C). As anticipated, HBsAg secretion was suppressed by the extra L protein construct but increased by the extra S protein construct (Figure 6B,C, bottom panels). The L protein construct reduced virion secretion in a dose-dependent manner (Figure 6B, 2nd and 3rd panels), while the S construct abolished virion secretion even at the lowest dose (Figure 6C, 2nd and 3rd panels). These results suggest that the L/S protein ratio achieved in the 1.1mer construct is optimal or nearly optimal for virion secretion; further increase or reduction in L or S protein expression will rather reduce virion secretion.

## 4. Discussion

HBV genotypes B and C co-circulate in East Asia and target hosts of similar ethnic backgrounds. Moreover, chronic infection by both genotypes is primarily attributed to perinatal transmission from HBeAg-positive mothers. However, genotype C isolates are more likely implicated in breakthrough infections of newborns despite combined active/passive immunization. Moreover, adulthood infection with genotype C has greater risk to become chronic [13]. On the other hand, genotype B infection is associated with a higher risk of fulminant hepatitis and acute exacerbation of chronic infection [14,15,16], and genotype B isolates respond to interferon therapy more favorably than genotype C isolates. Independent studies demonstrated that genotype C patients seroconvert from HBeAg to anti-HBe about 10 years later than genotype B patients, and, consequently, the prolonged active viral DNA replication and protein expression increase the lifelong risk for liver cirrhosis and HCC [7,8,9,10,11,12]. Based on our studies, wild-type genotype C isolates show a more reduced replication capacity than genotype B isolates but more efficient virion secretion [17]. We propose that the low replication capacity of wild-type genotype C isolates, meaning low expression levels of CORE protein, a strong immune target, may mitigate immune attack and delay HBeAg seroconversion. The low replication capacity may also serve as a driving force for the emergence of core promoter mutations during the immune clearance phase, which increase genome replication [26]. Indeed, genotype C isolates are more likely to develop core promoter mutations than genotype B isolates [8], which serve as independent risk factors for HCC development. The higher virion secretion efficiency of genotype C most likely compensates for its low replication capacity to promote virus transmission, both between different hosts and among hepatocytes inside the same liver. This will promote the establishment of persistent infection without triggering too strong an immune attack.

Our previous comparative study of the biological properties of genotype B and genotype C isolates was based on serum samples of Chinese and U.S. patients [17]. The full-length HBV genome was amplified by PCR and cloned to the *HindIII*-*SacI* sites of the pUC18 vector. All the genotype B isolates belonged to the B2 subgenotype, while the genotype C isolates were of the C2 subgenotype, except for a few C1 isolates. Two alternative approaches were taken afterwards. For the Chinese samples, the whole transformation product without plating was grown in liquid culture, followed by plasmid DNA preparation to generate a population pool. The 3.2-kb HBV genome was released from plasmid DNA by *BspQI* digestion, gel purified, and recircularized to mimic cccDNA. For the U.S. samples, individual PCR clones were converted to *SphI* dimer. Transient transfection experiments using both types of HBV DNA revealed the higher replication capacity of genotype B isolates/clones without core promoter mutations compared to the corresponding genotype C isolates/clones. Virion secretion was analyzed for the population pools (Chinese samples), with genotype C isolates showing much more efficient secretion (extracellular virion DNA/intracellular replicative DNA) than genotype B isolates.

The present study, as a direct extension of our reported work, was aimed at identifying the structural basis for differential virion secretion by the two HBV genotypes. Since the clone pools from the Chinese samples would complicate mutational analysis, we resorted to the individual clones from the U.S. samples with well-defined replication phenotype. Two clones of the B2 subgenotype (22.5 and 24.6) and one clone each of the C1 and C2 subgenotypes (27.2 and 17.3) were selected for the generation of chimeric constructs and site-directed mutants. Among the four clones, 22.5 and 24.6 have high (and comparable) replication capacities, while 17.3 has the lowest replication capacity [17]. Besides the *SphI* dimer, we also employed 1.1mer construct to markedly increase genome replication and, consequently, virion secretion. While that approach largely elevated the amount of intracellular replicative DNA for clone 27.2, clone 17.3 continued to display much less replicative DNA than the two genotype B clones (Figure 1A; Supplementary Figure S1A). Furthermore, some chimeric constructs showed greatly altered levels of replicative DNA than did the parental construct; thus, we had to resort to the ratio of virion DNA/replicative DNA as an indicator of virion secretion efficiency. We primarily used the Huh7 cell line to characterize the biological properties of these two HBV genotypes due to its higher transfection efficiency compared to HepG2 cells. Nevertheless, some key findings were further tested in HepG2 cells, and concordant results were obtained.

For both the 1.1mer and dimer constructs, clone 17.3 (C2) was most effective at virion secretion, followed by clone 27.2 (C1). In contrast, clone 22.5 had the lowest secretion efficiency (Figure 1, Figure 4, and Figure 6). Similar results were obtained in the HepG2 cell line. Functional characterization of additional clones of the C1 and C2 subgenotypes is needed to establish whether the C2 subgenotype has more efficient virion secretion than the C1 subgenotype. Reciprocal fragment exchange was then carried out between clones 17.3 and 24.6 and also between 27.2 and 22.5. We found that replacing the *core* gene between clones of the two HBV genotypes did not alter the efficiency of virion secretion (data not shown). In contrast, exchanging the entire *envelope* gene or just the *S* region between 17.3 and 24.6 could largely reverse the virion secretion phenotype (Figure 1C). Therefore, the *S* region, and probably also the *preS* region, harbor determinants for differential virion secretion. Similarly, exchanging the *S* region could increase virion secretion for clone 22.5, while diminishing virion secretion for clone 27.2. However, for unknown reasons, exchanging the entire *envelope* gene diminished virion secretion for 22.5 (Figure 1C, lane 2). One possibility is the incompatibility between the *preS* region and the *core* gene of these two clones. Considering that, with the 1.1mer construct, virion secretion was highest for clone 17.3 but lowest for clone 22.5, it will be interesting to generate chimeric constructs between these two clones to more effectively identify the determinants of differential virion secretion.

The L protein is engaged in core particle interaction, as well as the retention of the S protein for participation in virion morphogenesis, while the S protein is primarily responsible for particle (subviral particle and virion) formation and release. The M protein is not essential for virion secretion, but loss of M protein expression reduces the efficiency of virion secretion while increasing the maturity of the genome inside virions [22]. The *preS1*, *preS2*, and *S* domains of most HBV genotypes contain 119, 55, and 226 residues, respectively, of which 12, 12, and 20 positions are divergent between genotype B and genotype C isolates (Table 1). Previous studies identified a linear sequence at the boundary of the *preS1* and *preS2* domains (residues 103–124) as responsible for interaction with core particles [30,31]. Within that linear sequence, two residues are different between the two HBV genotypes; L108 and T115 for genotype B versus I108 and S115 for genotype C. Site-directed mutagenesis of both genotype B clones and both genotype C clones clearly demonstrated higher efficiency of I108/S115 than L108/T115 in mediating virion formation or release (Figure 4; Supplementary Figure S2). Interestingly, genotype A is similar to genotype C in having I108/S115, whereas genotype D has the same sequences as genotype B. Whether such a difference affects virion secretion for genotypes A and D remains to be determined.

Twenty residues in the S domain are divergent between the two genotypes (Table 1). The current study focused on residue 126 for several reasons. First, genotype C is unique in having I126 rather than T126, which is found in other HBV genotypes. Second, residue 126 is part of the so-called ‘a’ determinant (residues 124–147), which is exposed on the virion surface and constitutes a major target of neutralizing antibodies. Indeed, immune escape mutations such as G145R frequently arise during the late stages of HBV infection or in association with vaccine escape or occult HBV infection [37,38,39,40]. In this regard, residue 126 is frequently mutated during the late stage of HBV infection, such as I126T/S/N for genotype C and T126A for other HBV genotypes [41,42]. There was a report that the I126S mutation can cause decreased HBsAg detection and occult HBV infection [43]. Third, many immune escape mutants are impaired in virion secretion [37,43,44]. Our transfection experiments confirmed that I126 supported virion secretion from genotype C clones better than T126, although whether the T126I mutation improves virion secretion for genotype B remains inconclusive (Figure 5C). We also observed the association of I126 with a higher HBsAg titer than T126 in culture supernatant (Figure 5D), which is consistent with the result in a recent study [41]. Certainly, whether this is genuine or an artifact of the preferred recognition of I126 over T126 by our ELISA kit remains to be clarified.

In our initial study, the higher replication capacity of genotype B isolates/clones compared to genotype C isolates/clones correlated with higher level of the 3.5-kb RNA, suggesting control at the transcriptional level [17]. In the follow-up investigation, genotype B clones were found to possess a stronger enhancer II and/or core promoter region (these two elements overlap), and replacement of that region (1627–1866) was sufficient to reverse the replication phenotype [45]. In this regard, it was quite unexpected that replacement of the *envelope* gene or the *S* region of the 1.1mer construct of clone 17.3 with that of clone 24.6 could markedly increase the level of intracellular replicative DNA, whereas the reverse exchange could greatly reduce replication for 24.6 (Figure 1A, left panel). Moreover, similar findings can be made with *SphI* dimer, which represents a more physiological form of HBV DNA (Figure 1A, right panel). Similarly, replacement of the *envelope* gene of clone 22.5 with that of clone 27.2 suppressed replicative DNA for both 1.1mer and dimer constructs. However, enhancer I, enhancer II, or core promoter, which can all regulate levels of the 3.5-kb pgRNA, lie outside the *envelope* gene. Northern blot analysis or primer extension assay will be needed to establish whether the altered level of replicative DNA correlates with a changed level of the 3.5-kb RNA or pgRNA.

It is worth mentioning that clone 17.3, which showed the lowest level of intracellular replicative DNA in the 1.1mer form, had the highest efficiency of virion secretion. A similar correlation can be made for the chimeric constructs; those with increased intracellular replicative DNA had reduced virion secretion and vice versa. Thus, one intriguing possibility is that increased interaction between core particles and HBV ENVELOPE proteins will commit such particles towards the secretory pathway, although a large fraction will be degraded rather than secreted. The outcome is both a high level of virion DNA and a low level of intracellular replicative DNA. It will be very interesting to determine whether preventing the expression of ENVELOPE proteins from the 1.1mer genome of clone 17.3 or chimeric constructs with altered replication capacity will revert the phenotype of replicative DNA and whether suppressing the proteasome or lysosome degradation pathway can increase level of intracellular HBV DNA for clones with ‘low replication’ phenotypes such as 17.3.

Another interesting observation is that the replacement of the entire *envelope* gene of the two genotype B clones with genotype C sequences also reduced HBsAg in culture supernatant (Figure 3B). The opposite effect was seen when the *envelope* gene of the two genotype C clones was replaced with that of genotype B, although to lesser extent. No such effect was seen when just the *S* region was exchanged. It will be very helpful to measure the HBsAg titer from cell lysate to calculate the extracellular/intracellular HBsAg ratio to determine whether HBsAg production or its secretion was diminished. If HBsAg production was reduced, then genotype C isolates may have a weaker SPII promoter, which directs the transcription of the 2.1-kb RNA for M/S proteins and is located in the *preS* region. Indeed 28 positions within the SPII promoter are divergent between genotypes B and C (data not shown). If HBsAg secretion was reduced, then the L protein of genotype C might be more efficient at retaining the S protein (and possibly promoting virion formation through the same mechanism).

## Figures and Tables

**Table 1:** Divergent amino acid (aa) positions between genotype B and genotype C isolates in the *preS1*, *preS2*, and *S* domains.

***preS1*** **aa Position**												
	10	35	39	45	48	51	54	57	60	84	108	115
Genotype B (2246 isolates)	K	K	E	L	H	N	D	K	V	L	L	T
Genotype C (2227 isolates)	Q	G	N	F	N	H	E	Q	A	I	I	S
***preS2*** **aa Position**												
	130	132	138	152	154	155	156	158	160	165	167	172
Genotype B (2419 isolates)	T	Q	A	S	A	Q	N	V	A	L	K	V
Genotype C (2377 isolates)	A	L	G	N	V	P	T	A	P	F	R	A
***S*** **aa Position**												
	4	5	8	24	45	47	49	56	57	59		
Genotype B (4224 isolates)	I	A	L	K	T	V	L	Q	I	S		
Genotype C (7183 isolates)	T	T	F	R	A	T	P	P	T	N		
	64	85	110	113	126	143	160	161	200	213		
Genotype B (4224 isolates)	C	C	I	S	T	T	K	Y	F	M		
Genotype C (7183 isolates)	S	F	L	T	I	S	R	F	Y	L		

## Supplementary Materials

The supplementary materials are available online at www.mdpi.com/1999-4915/9/4/62/s1. Figure S1: Comparison of HBV genome replication and virion secretion between genotype B and C clones in two different human hepatoma cell lines; Figure S2: Impact of exchanging the entire *S* region or positions 108 and 115 in the *preS1* region between genotype B and C clones on genome replication and virion secretion: results from HepG2 cells.
