# Supplementary material for: The Envelope Gene of Hepatitis B Virus Is Implicated in Both Differential Virion Secretion and Genome Replication Capacities between Genotype B and Genotype C Isolates
Source: Viruses. 2017 Mar 28;9(4):62. doi: 10.3390/v9040062 (PMC5408668; doi:10.3390/v9040062)
Supplement: Supplementary file 1 [file viruses-09-00062-s001.pdf]

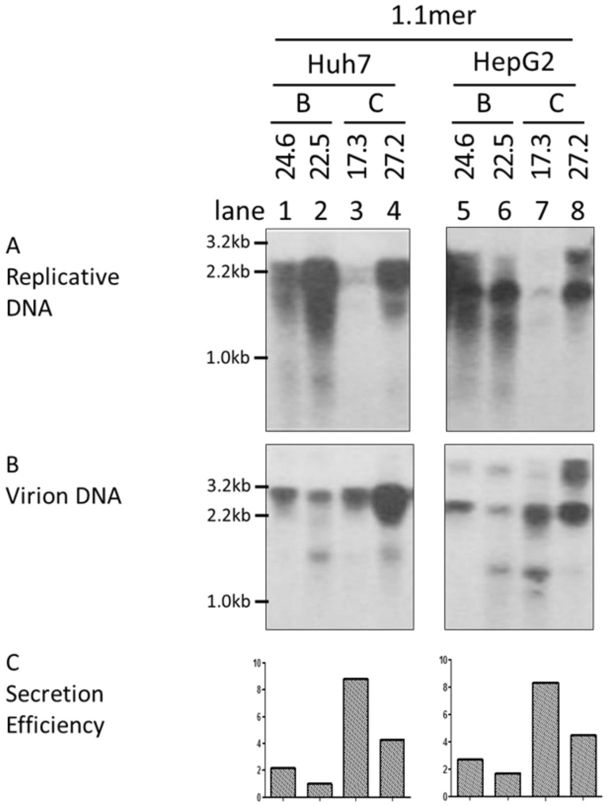

Supplementary Fig. 1. Comparison of HBV genome replication and virion secretion between genotype B and C clones in two different human hepatoma cell lines.
1.1mer construct of the two genotype B clones (24.6 and 22.5) and two genotype C clones (17.3 and 27.2) was transiently transfected to Huh7 cells (left panels) or HepG2 cells (right panels), using 2µg DNA per well of the 6-well plates. Shown are Southern blot analysis of replicative DNA in cell lysate (A) and virion DNA from cultured supernatant (B). Virions were immunoprecipitated by anti-preS1 and anti-S antibodies, and the blots were hybridized with mixed probes of the two HBV genotypes. (C) Calculated ratio of extracellular virion DNA/intracellular replicative DNA following densitometric analysis of the Southern blots using ImageJ software. The value for clone 22.5 of genotype B was set arbitrarily at 1.

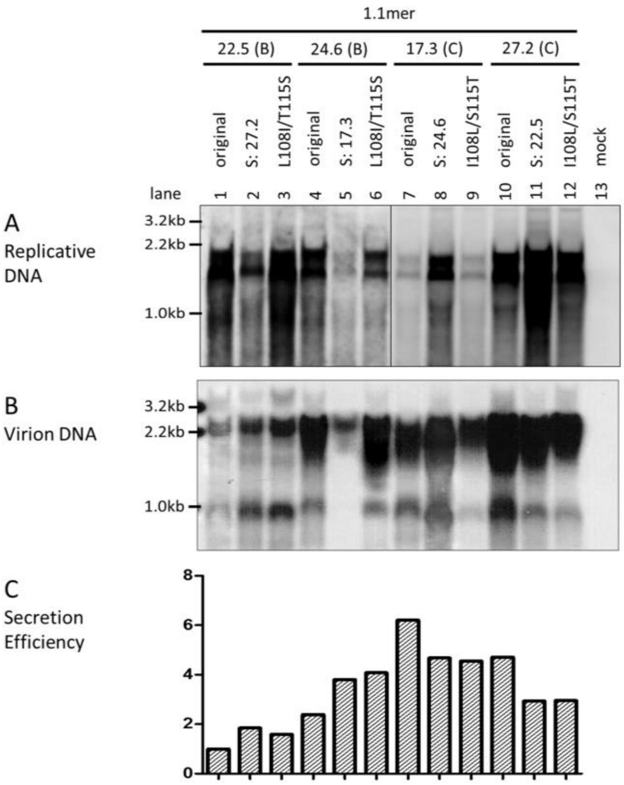

Supplementary Fig. 2. Impact of exchanging the entire S region or positions 108 and 115 in the preS1 region between genotype B and C clones on genome replication and virion secretion: results from HepG2 cells. The genotype B clones have L108 and T115 in the preS1 domain in contrast to I108 and S115 found in genotype C. Either the S region was swapped between the two genotype B clones and two genotype C clones or positions 108 and 115 in the preS1 region were mutated to those of the other genotype. The 1.1mer genomes of the original construct, S region chimera, and site-directed mutant were transfected to HepG2 cells, which were harvested 4 days later. (A) Southern blot analysis of intracellular replicative DNA. (B) Southern blot analysis of extracellular virion DNA. (C) Ratio of extracellular virion DNA/intracellular replicative DNA. Densitometric values were obtained from Southern blots using ImageJ software. The ratio for clone 22.5 was set arbitrarily at 1.
